# Evaluation of the susceptibility of *Alphitobius diaperinus* meal to infestations by major stored-product beetle species

**DOI:** 10.1007/s11356-023-27602-0

**Published:** 2023-05-17

**Authors:** Marianna Rigopoulou, Christos Rumbos, Christos Athanassiou

**Affiliations:** Laboratory of Entomology and Agricultural Zoology, Department of Agriculture, Crop Production and Rural Environment, University of Thessaly, Phytokou Str, 38446 Volos, Magnesia Greece

**Keywords:** Alternative proteins, Insect infestation, Insect meal, Population growth, Stored-product insects

## Abstract

The projections for the production of insects as food and feed show an enormous increase for insect production in the near future, which will subsequently lead to the increase of the stored quantities of insect meals and related products. However, information on the susceptibility of insect meals to infestations by stored-product insects is rather limited. To this end, the objective of the present study was to evaluate the potential of major storage insect species to grow and reproduce on insect meals that are based on larvae of the lesser mealworm, *Alphitobius diaperinus*. The progeny production of thirteen stored-product insects on *A. diaperinus* meal, as well as their instantaneous rate of increase, as a measure of population growth, was recorded for each species. Based on the results, six out of the thirteen examined insect species (*A. diaperinus*, *Tenebrio molitor*, *Trogoderma granarium*, *Lasioderma serricorne*, *Tribolium confusum*, and *Tribolium castaneum*) were able to infest pure *A. diaperinus* meal, as they grew well and developed progeny on the insect meal substrate. *Tribolium confusum*, *T. castaneum*, and especially *T. granarium* gave the highest progeny production numbers in the *A. diaperinus* meal with the latter giving an instantaneous rate of increase of 0.067. Expecting the upcoming increase in the production of insect-based products globally, further research in this field is needed for improved production and storage facilities, detection and estimation methods, and technologies to minimize insect infestations without causing negative effects to farmed insects.

## Introduction

The challenge to feed the increasing world’s population has led to excessive production and prolonged storage of food and feed with the preservation of their quantity and quality being at risk. Stored-product insects seem to be the major problem of all kinds of durable agricultural products during their storage with a wide range of insect species leading to considerable losses and qualitative degradations (Phillips and Throne 2010; Mason and Mcdonald 2012; Nayak and Daglish 2018). The enemies of stored products are already categorized according to their biology and food preferences. According to this, some of the major pests are primary colonizers and have the capacity to infest sound grain kernels, such as the species of genus *Sitophilus* (Coleoptera: Curculionidae), e.g., the granary weevil, *Sitophilus granarius* (L.), and the rice weevil, *Sitophilus oryzae* (L.), or of the genus *Callosobruchus* (Coleoptera: Bruchidae), such as the cowpea weevil, *Callosobruchus maculatus* (F.) (Rees 2007; Hagstrum et al. 2012; Athanassiou and Arthur 2018). Other species are classified as secondary colonizers, which are prone to infest processed commodities, or commodities that are already infested by primary colonizers, such as species of the genus *Tribolium* (Coleoptera: Tenebrionidae), e.g., the red flour beetle, *Tribolium castaneum* (Herbst), and the confused flour beetle, *Tribolium confusum* Jacquelin du Val (Nayak and Daglish 2018; Athanassiou and Arthur 2018; Campbell et al. 2022).

At the same time, the increasing global need for food and feed adequacy led to finding alternative nutritional sources with insects showing great promise towards this end (FAO 2013; Van Huis 2013; Kelemu et al. 2015; Yen 2015; Dossey et al. 2016; Rumbos and Athanassiou 2021). Although in its early steps, the massive production of insect meals and insect-based products has become a noticeable part of the production chain during the last few years due to the utilization of registered species for this purpose, such as the yellow mealworm, *Tenebrio molitor* L. (Coleoptera: Tenebrionidae), and the lesser mealworm, *Alphitobius diaperinus* Panzer (Coleoptera: Tenebrionidae) (Sánchez-Muros et al. 2014; Van Huis et al. 2015; Commission Regulation (EU) 2017/893; 2021/1372; Van Huis 2019; EFSA 2022). Furthermore, predictions show an enormously increasing insect production in the near future (Meticulous Market Research 2022).

The increase in the production of insect products demands a subsequent increase in the need for their storage. Thus, the edible insect market has to deal with all the problems the conventional agricultural products face during their storage including insect infestations that usually infest durable agricultural commodities (Rumbos et al. 2020a; Deruytter et al. 2021). Earlier work indicates that certain stored-product insect species can feed upon dead insect individuals (Hagstrum et al. 2012), but the degree that this infestation can be detrimental to insect meals is poorly understood. In an earlier study, Rumbos et al. (2020a) studied the vulnerability of insect-based substrates that are based on *T. molitor* to insect infestations and have shown that certain species can easily develop in such substrates including *T. molitor* itself. Hence, from a practical view, the increase of the storage capacity and duration of insect-based meals may concomitantly increase “post-harvest” infestations and losses by stored-product arthropods.

Despite these initial reports, the data available so far are focused only on a narrow range of stored-product insect species, while there is still inadequate information for the vast majority of the most common species that occur in durable agricultural commodities. The evaluation of a high number of stored-product insect species on insect meal substrates would be crucial for specifying their ability to infest stored products and food based on insects. This evaluation is also essential to draw the inferences necessary for control strategies that are species-specific, considering that some species that infest these substrates can be controlled easier than others (Deruytter et al. 2021). For instance, control of the Indian meal moth, *Plodia interpunctella* (Hübner) (Lepidoptera: Pyralidae), can be done with the application of mating disruption, which avoids conventional chemical control but is not possible for most stored-product beetle species (Burks et al. 2011; Deruytter et al. 2021). A species-specific strategy for the control of insect infestations in facilities that produce insect meals is much more complicated in comparison with that in flour or feed mills, considering that certain control measures can also kill the “beneficial” insects (Rumbos et al. 2020a). In this context, we have evaluated the potential of thirteen common stored-product insect species to grow and reproduce on insect meals that are based on *A. diaperinus* larvae. In this effort, we have included species that range from primary to secondary colonizers, as well as species that can be classified either as stenophagous or polyphagous, to test their ability to constitute a risk in stored insect-based commodities.

## Materials and methods

### Insect rearing

All insect species used in the tests were reared at the Laboratory of Entomology and Agricultural Zoology, Department of Agriculture, Crop Production and Rural Environment, University of Thessaly. The thirteen species tested here were *S. oryzae*; *S. granarius*; *C. maculatus*; *T. castaneum*; *T. confusum*; *T. molitor*; *A. diaperinus*; the sawtoothed grain beetle, *Oryzaephilus surinamensis* (L.) (Coleoptera: Silvanidae); the khapra beetle, *Trogoderma granarium* Everts (Coleoptera: Dermestidae); the lesser grain borer, *Rhyzopertha dominica* (F.) (Coleoptera: Bostrychidae); the larger grain borer, *Prostephanus truncatus* (Horn) (Coleoptera: Bostrychidae); the cigarette beetle, *Lasioderma serricorne* (F.) (Coleoptera: Anobiidae), and the rusty grain beetle, *Cryptolestes ferrugineus* (Stephens) (Coleoptera: Laemophloeidae). Each species was reared on its preferred diet; namely, *S. oryzae*, *S. granarius*, *T. granarium*, and *R. dominica* were reared on soft wheat kernels, while *T. castaneum* and *T. confusum* were reared on white wheat flour (Loulis Mills, Organic category M soft wheat flour). *Callosobruchus maculatus* was reared on chickpeas (Tyrnavos Food S.A., Tyrnavos, Greece), *O. surinamensis* on oat flakes (Quaker Oats Company, Chicago, Illinois, USA), and *P. truncatus* on maize grains (bought from a local retailer). For *L. serricorne*, a mixture of maize flour and yeast (Angel Yeast Co. Ltd., Yichang, China) (18:1) was used, and for *A. diaperinus*, a mixture of wheat bran (bought from a local retailer) and egg layer hen pellets (3:1) (No 3–Compound Feed for Layers, Viozokat S.A., Katerini, Greece). *Tenebrio molitor* and *C. ferrugineus* were reared using wheat bran. *Tenebrio molitor* and *A. diaperinus* were weekly supplied with fresh potatoes and apples (bought from a local store), respectively, as a moisture source. Except for *A. diaperinus* and *T. granarium* which were kept at 30 °C and 55% relative humidity (r.h.) and 32 °C and 55% r.h., respectively, all the other species were kept at 26 °C and 55% r.h. All insect species were kept at continuous darkness. Only adults, < 1 month old, were used in the tests.

### Insect meal preparation

For the *A. diaperinus* meal preparation, late-stage larvae were used after “harvesting” them through separation from the feeding substrate with sieving. The larvae were frozen at – 20 °C, chopped using a stainless-steel mill (Thermomix TM31-1, Vorwerk Elektrowerke GmbH & Co. KG, Wuppertal, Germany), dried for 72 h at 60 °C, and finally sieved with a 1-mm opening sieve. The insect meal produced was stored at − 20 °C until the initiation of the tests.

### Experimental design

The population growth of each of the thirteen species was evaluated on pure *A. diaperinus* meal substrate (100% *A. diaperinus* meal). The substrates used as the control reference were those used for the rearing of each species described above. The whole experimental procedure was conducted in cylindrical plastic vials (Rotilabo®-sample tins with snap-on lid, 3.0 cm in diameter, 8.0 cm in height, Carl Roth GmbH & Co. Kg, Karlsruhe, Germany), on which an opening (1.5 cm in diameter) was created in the lid and covered with muslin gauze in order to ensure proper aeriation of the vial. Polytetrafluoroethylene preparation (Fluon, 60 wt% dispersion in water, Sigma-Aldrich Chemie GmbH, Steinheim, Germany) was applied on the upper and inner part of the vials to prevent insects from escaping the vials. Five grams of each of the mentioned substrates was placed in the vials, using different vials for each substrate. Finally, twenty mixed-sex adults of each of the thirteen insect species tested were placed into the vials, using different series of vials for each species. All vials were then placed at the conditions mentioned above, proper for each species, and were kept there for 65 days. In the case of *T. molitor* and *A. diaperinus*, a slice of potato and carrot, respectively, was placed inside the vials twice a week to cover the insect moisture needs. For all species, after 65 days, the vials were opened for the evaluation of the progeny production, through counting separately the total number of adults (alive or dead) and larvae. For *O. surinamensis*, *L. serricorne*, *T. confusum*, and *T. castaneum*, the number of pupae was also determined, whereas for *A. diaperinus* and *T. molitor*, the total larval weight was also recorded. For the latter species, the average individual larval weight was also calculated by dividing the total larval weight by the total number of the larvae. As a direct measure of population growth, for all species, the instantaneous rate of increase was calculated using the following equation: *r*_i_ = ln(*N*_f_/*N*_o_)/Δ*T*, where *N*_f_ was the final number of individuals, *N*_o_ was the initial number of individuals, and Δ*T* was the change in time, i.e., the duration of the experiment. Positive values of *r*_i_ suggest a growing population, *r*_i_ = 0 shows a stable population, and negative *r*_i_ values specify a population in decline (Stark and Banks 2003). There were three replicates for each treatment (three-vial replicates) with the whole procedure to be repeated two times (two series of vials) by preparing new vials each time (3 × 2 = 6 vials for each combination).

### Statistical analysis

At first, the data were checked for normality using Shapiro–Wilk’s test (Zar 1999). For all species tested, the data for the different life stages that were evaluated, as well as the instantaneous rates of increase, were tested for normality and found that they were non-normally distributed. Therefore, data were compared with the Kruskal–Wallis *H*-test followed by multiple pairwise Mann–Whitney *U* tests using the Bonferroni correction (Zar 1999). All analyses were conducted using the IBM® SPSS® Statistics software, Version 25 (IBM Corporation, Armonk, NY, USA).

## Results

From all the insect species tested in the bioassays, *S. oryzae*, *S. granarius*, *R. dominica*, *P. truncatus*, *O. surinamensis*, *C. maculatus*, and *C. ferrugineus* did not manage to grow and develop on pure *A. diaperinus* meal (100% insect meal). Hence, for these species, there was no progeny production on *A. diaperinus* substrate, since the total number of individuals found at the termination of the tests was the twenty initially inserted adults, while in some cases, less than twenty adults were recorded (Tables 1, 2, 3, and 4). From the species tested, *T. granarium* and *T. castaneum* gave that highest progeny production in the control substrates among the species tested, which was 1570 and 445 individuals/vial, respectively, after 65 days (Tables 4 and 5) with *T. granarium* giving an instantaneous rate of increase of 0.067 (Table 4).

**Table 1:** Population growth (mean number of individuals (± SE) per vial) and instantaneous rate of increase of *Sitophilus oryzae*, *Sitophilus granarius*, *Rhyzopertha dominica*, and *Prostephanus truncatus* after 65 days on 100% *Alphitobius diaperinus* meal and on control (soft wheat grains (*S. oryzae*, *S. granarius*, *R. dominica*); maize grains (*P. truncatus*))

	Substrate	*Sitophilus oryzae*	*Sitophilus granarius*	*Rhyzopertha dominica*	*Prostephanus truncatus*
Adults (dead)	Control	18.0 ± 1.7	38.3 ± 3.0*	8.5 ± 3.7	4.2 ± 1.4*
	100% *A. diaperinus* meal	19.3 ± 0.2	20.0 ± 0.0	17.2 ± 0.3	19.7 ± 0.2
Adults (alive)	Control	58.2 ± 14.6*	22.7 ± 4.9*	21.7 ± 3.9*	15.7 ± 1.9*
	100% *A. diaperinus* meal	0.0 ± 0.0	0.0 ± 0.0	0.0 ± 0.0	0.0 ± 0.0
Total number of individuals	Control	76.2 ± 13.8*	61.0 ± 6.5*	30.2 ± 5.6*	19.8 ± 3.0
	100% *A. diaperinus* meal	19.3 ± 0.2	20.0 ± 0.0	17.2 ± 0.3	19.7 ± 0.2
Instantaneous rate of increase	Control	0.019 ± 0.0028*	0.0167 ± 0.0020*	0.0054 ± 0.0025*	0.0013 ± 0.0013
	100% *A. diaperinus* meal	0.0 ± 0.0	0.0 ± 0.0	0.0 ± 0.0	0.0 ± 0.0

Within each column, control means followed by an asterisk (*) are significantly different from those on *A. diaperinus* meal (in all cases, df = 1, 11; Mann–Whitney *U* test at 0.05). Where no asterisk exists, no significant differences were noted

**Table 2:** Population growth (mean number of individuals (± SE) per vial) and instantaneous rate of increase of *Alphitobius diaperinus* and *Tenebrio molitor* after 65 days on 100% *A. diaperinus* meal and on control (mixture of wheat bran and egg layer hen pellets (3:1) (*A. diaperinus*); wheat bran (*T. molitor*))

	Substrate	*Alphitobius diaperinus*	*Tenebrio molitor*
Adults (dead)	Control	1.7 ± 0.4	4.7 ± 1.6
	100% *A. diaperinus* meal	2.0 ± 0.4	4.8 ± 2.1
Adults (alive)	Control	17.7 ± 0.5	14.3 ± 2.0
	100% *A. diaperinus* meal	18.0 ± 0.4	13.0 ± 2.8
Larvae	Control	376.3 ± 44.7	113.8 ± 7.4
	100% *A. diaperinus* meal	390.5 ± 37.0	174.7 ± 50.9
Average individual larval weight (mg)	Control	8.5 ± 0.8*	16.9 ± 1.3*
	100% *A. diaperinus* meal	15.7 ± 1.2	33.5 ± 7.4
Total number of individuals	Control	395.7 ± 44.7	130.5 ± 7.4
	100% *A. diaperinus* meal	410.5 ± 37.0	191.7 ± 50.1
Instantaneous rate of increase	Control	0.0454 ± 0.0017	0.0287 ± 0.0009
	100% *A. diaperinus* meal	0.0461 ± 0.0016	0.0317 ± 0.0046

Within each column, control means followed by an asterisk (*) are significantly different from those on *A. diaperinus* meal (in all cases, df = 1, 11; Mann–Whitney *U* test at 0.05). Where no asterisk exists, no significant differences were noted

**Table 3:** Population growth (mean number of individuals (± SE) per vial) and instantaneous rate of increase of *Oryzaephilus surinamensis*, *Callosobruchus maculatus*, and *Lasioderma serricorne* after 65 days on 100% *Alphitobius diaperinus* meal and on control (oat flakes (O. s*urinamensis*); chickpeas (*C. maculatus*); mixture of maize flour and yeast (18:1) (*L. serricorne*))

	Substrate	*Oryzaephilus surinamensis*	*Callosobruchus maculatus*	*Lasioderma serricorne*
Adults (dead)	Control	6.5 ± 0.8*	144.8 ± 8.8*	37.3 ± 3.8
	100% *A. diaperinus* meal	12.2 ± 0.5	20.0 ± 0.4	12.3 ± 2.1
Adults (alive)	Control	179.8 ± 14.7*	0.0 ± 0.0	83.1 ± 50.2*
	100% *A. diaperinus* meal	4.7 ± 0.5	0.0 ± 0.0	111.5 ± 23.1
Larvae	Control	40.0 ± 7.2*	-	273.2 ± 88.2
	100% *A. diaperinus* meal	0.0 ± 0.0	-	124.5 ± 30.3
Pupae	Control	2.3 ± 0.6*	23.3 ± 5.0*	4.3 ± 3.4*
	100% *A. diaperinus* meal	0.0 ± 0.0	0.0 ± 0.0	41.8 ± 18.7
Total number of individuals	Control	228.7 ± 18.2*	168.2 ± 10.7*	398.0 ± 44.5
	100% *A. diaperinus* meal	16.8 ± 0.5	20.0 ± 0.4	290.2 ± 23.0
Instantaneous rate of increase	Control	0.0372 ± 0.0012*	0.0326 ± 0.0011*	0.0455 ± 0.0018
	100% *A. diaperinus* meal	0.0 ± 0.0	0.0 ± 0.0	0.0409 ± 0.0012

Within each column, control means followed by an asterisk (*) are significantly different from those on *A. diaperinus* meal (in all cases, df = 1, 11; Mann–Whitney *U* test at 0.05). Where no asterisk exists, no significant differences were noted

**Table 4:** Population growth (mean number of individuals (± SE) per vial) and instantaneous rate of increase of *Trogoderma granarium* and *Cryptolestes ferrugineus* after 65 days on 100% *Alphitobius diaperinus* meal and on control (soft wheat grains (*T. granarium*); wheat bran (*C. ferrugineus*))

	Substrate	*Trogoderma granarium*	*Cryptolestes ferrugineus*
Adults (dead)	Control	12.8 ± 5.8*	0.5 ± 0.2*
	100% *A. diaperinus* meal	165.7 ± 10.0	20.0 ± 0.0
Adults (alive)	Control	2.8 ± 1.8	356.0 ± 29.4*
	100% *A. diaperinus* meal	2.0 ± 1.6	0.0 ± 0.0
Larvae	Control	311.3 ± 48.8*	45.3 ± 4.4*
	100% *A. diaperinus* meal	1402.8 ± 85.8	0.0 ± 0.0
Total number of individuals	Control	327.0 ± 50.7*	401.8 ± 31.4*
	100% *A. diaperinus* meal	1570.5 ± 84.9	20.0 ± 0.0
Instantaneous rate of increase	Control	0.0420 ± 0.0025*	0.0459 ± 0.0013*
	100% *A. diaperinus* meal	0.0670 ± 0.0008	0.0 ± 0.0

Within each column, control means followed by an asterisk (*) are significantly different from those on *A. diaperinus* meal (in all cases, df = 1, 11; Mann–Whitney *U* test at 0.05). Where no asterisk exists, no significant differences were noted

**Table 5:** Population growth (mean number of individuals (± SE) per vial) and instantaneous rate of increase of *Tribolium confusum* and *Tribolium castaneum* after 65 days on 100% *Alphitobius diaperinus* meal and on flour (control)

	Substrate	*Tribolium confusum*	*Tribolium castaneum*
Adults (dead)	Control	1.2 ± 0.5*	2.5 ± 1.0*
	100% *A. diaperinus* meal	2.0 ± 0.6	13.0 ± 3.7
Adults (alive)	Control	28.7 ± 2.5*	22.0 ± 1.2*
	100% *A. diaperinus* meal	204.7 ± 15.2	374.3 ± 44.7
Larvae	Control	16.7 ± 2.2*	9.5 ± 1.7*
	100% *A. diaperinus* meal	103.0 ± 9.0	56.7 ± 9.9
Pupae	Control	8.0 ± 1.3*	1.0 ± 0.4
	100% *A. diaperinus* meal	79.0 ± 7.3	1.2 ± 0.7
Total number of individuals	Control	54.5 ± 3.6*	35.0 ± 1.4*
	100% *A. diaperinus* meal	388.7 ± 26.0	445.2 ± 41.4
Instantaneous rate of increase	Control	0.0131 ± 0.0028*	0.0085 ± 0.0006*
	100% *A. diaperinus* meal	0.0455 ± 0.0011	0.0474 ± 0.0013

Within each column, control means followed by an asterisk (*) are significantly different from those on *A. diaperinus* meal (in all cases, df = 1, 11; Mann–Whitney *U* test at 0.05). Where no asterisk exists, no significant differences were noted

The rest of the examined species grew and developed on 100% *A. diaperinus* meal, giving a total progeny production ranging from 192 individuals (*T. molitor*) to 410 individuals (*A. diaperinus*) (Table 2) and instantaneous rates of increase ranging between 0.0317 and 0.0461 (Tables 2, 3, and 5). These numbers were found to be significantly higher than the control in most of the cases tested, with the exception of *A. diaperinus* and *T. molitor*, in which no significant differences were found, apart from their average individual larval weight (Table 5).

## Discussion

Our results showed clearly that six out of the thirteen examined insect species in this study can indeed infest pure *A. diaperinus* meal. Particularly, *A. diaperinus*, *T. molitor*, *T. granarium*, *L. serricorne*, *T. confusum*, and *T. castaneum* grew well and produced progeny on the insect meal substrate. Moreover, in some of the cases tested, progeny production in the control vials was comparable with that for the vials that contained *A. diaperinus* meal, suggesting that the latter is not just a marginal rearing substrate for some species, but a good nutrient source that can support their development. The fact that, under certain circumstances, insect meals can be easily infested by stored-product insects has been recently examined by Rumbos et al. (2000a), using *T. molitor* meal. Our data indicate that *A. diaperinus* meal was equally susceptible to infestations by stored-product insects, if not even more susceptible than that of *T. molitor*. This fact is particularly important, as stored-product insects can pose a risk in insect farming that will require control measures.

Although some of the species tested here were not able to reproduce on *A. diaperinus* meal, we think that these species can still be a threat to insect farming, through their occurrence in the raw materials that are used as rearing substrates. In the case of both *A. diaperinus* and *T. molitor*, these raw materials are mostly based on amylaceous commodities, such as bran (Ribeiro et al. 2018; Rumbos et al. 2020b; c; 2021), that can be easily infested by a wide range of species, such as *S. oryzae*, *S. granarius*, *R. dominica*, and *P. truncatus*, which are primary colonizers in grain kernels (Rees 2007; Hagstrum et al. 2012; Athanassiou and Arthur 2018), or *C. ferrugineus*, which can easily develop in a wide range of amylaceous products (Hagstrum et al. 2012; 2013). On the other hand, species like *C. maculatus* are stenophagous and can infest only a certain range of commodities, which may be less important in insect farming. Nevertheless, the introduction of these species into a given insect production facility is likely to occur though the raw materials and can be rapidly increased at the first stages of the rearing, when the diet is introduced in the insect rearing. On the other hand, their importance can be decreased at the later stage of the rearing, when the insect diet becomes frass. In this context, any control effort should give emphasis to the disinfestation of the raw materials, before their introduction to the insect production lines. Finally, it should be noted that the primary colonizers tested here cannot develop easily in cracked kernels (Hagstrum et al. 2012), so chopping/milling of the grains that are to be used in insect farming may provide a certain degree of protection against these species.

Not surprisingly, species that are secondary colonizers and have a preference for processed amylaceous commodities (Hagstrum et al. 2012; 2013; Nayak and Daglish 2018; Athanassiou and Rumbos 2018) could develop easily in *A. diaperinus* meal. This observation stands in accordance with previous results reported by Rumbos et al. (2020a) for some of the species tested here. We think that this group of species poses the most considerable risk in terms of insect infestation, as they can infest cracked grain kernels during the feeding process to produce *A. diaperinus* meals, and, as such, their presence is even more important at that stage, as compared with their presence to the stored raw materials (e.g., grains). This fact also complicates the application of control measures, because, even if the raw materials are disinfested, they can establish high populations in the processing stage and infest *A. diaperinus* meals throughout the entire production line. For instance, fumigation with phosphine has been proved to be effective for stored-product insect control in storage and processing facilities (Nayak et al. 2020), but could not be used in certain parts of the production line in insect farming, due to the effect of phosphine in the “beneficial” insects.

From the species tested here, we found that *T. confusum*, *T. castaneum*, and, especially, *T. granarium* gave the highest progeny production numbers in the *A. diaperinus* meals. The two species of the genus *Tribolium* can feed on dead insects (Campbell 1989; Hill 2002), although to a lesser extent in comparison with other stored-product beetle species, but their population growth is highly enhanced by processed products, such as flour (Hagstrum and Subramanyam 2009; Hagstrum et al. 2013), which can partially explain the progeny production levels noted here. Moreover, apart from the progeny production capacity in absolute numbers, our results clearly demonstrate that the presence of *A. diaperinus* meal increased the speed of development of *Tribolium* spp., providing a faster larval growth and adult emergence. On the other hand, *T. granarium* is widely known as a “dirty feeder” and can feed upon dead insects as well (Athanassiou et al. 2019). In an earlier study, testing the competition capacity of *T. granarium* over other major stored-product beetle species, Kavallieratos et al. (2017) found that, at increased temperatures, *T. granarium* could outcompete *S. oryzae* and *R. dominica*, and after a certain period of time, the vials that initially contained all three species contained only *T. granarium* individuals.

Interestingly, we found that *A. diaperinus* can develop easily in *A. diaperinus* meals. While this may not be a serious problem in the mass rearing facilities of this species, the infestation of *A. diaperinus* in the final product during storage and transportation is likely to cause serious losses, as apart from the main infestation per se, this species can transmit a wide range of bacteria that may endanger human and animal health (Rumbos et al. 2019). Thus, any “escapees” from the production line may invade the final product, causing serious degradations, while remaining undetectable. Similarly, certain stored-product species can transmit pathogens that are not food-borne and can induce antibiotic resistance, as in the case of *Tribolium* spp. that can transfer certain species of Enterococci (Channaiah et al. 2010; Hubert et al. 2018; Parlapani et al. 2020). In the same way, the occurrence of larvae of *T. granarium* in the final commodity may cause serious allergenic reactions including skin and eye irritations (Athanassiou et al. 2019).

To conclude, the present study illustrated the ability of major stored-product insects to infest commodities of pure *A. diaperinus* meal in a way comparable with the infestations of the conventional agricultural stored products. The impact of such infestations may be visible in certain qualitative characteristics of the final product or even during the production chain through cross infestations. Expecting the upcoming increase in the production of insect-based products globally, research on this field should be more focused on improved production and storage facilities, detection and estimation methods, and technologies to minimize insect infestations, without detrimental effects on the insects that are to be produced in insect farming units.

## Data Availability

The datasets used in this study are available upon reasonable request from the corresponding author.

## Declarations

### Disclaimer

The data and opinions expressed in this manuscript are solely our own, and we are solely responsible for their authenticity, validity, and originality. Any use of trade, firm, or product names is for descriptive purposes only and does not imply endorsement by the University of Thessaly.
